# Nm23-H1 suppresses hepatocarcinoma cell adhesion and migration on fibronectin by modulating glycosylation of integrin beta1

**DOI:** 10.1186/1756-9966-29-93

**Published:** 2010-07-11

**Authors:** Shangyang She, Boying Xu, Min He, Xiuwan Lan, Qiuyan Wang

**Affiliations:** 1 Clinical Laboratory, Guangxi Maternal and Child Health Hospital, Nanning 530003, China; 2 Huzhou Teacher's College Medical School, Huzhou 313000, China; 3 Guangxi Medical University, Nanning530021, China

## Abstract

**Background:**

Nm23 gene was isolated as a metastatic suppressor gene. The antimetastatic effect of Nm23 has been an enigma for more than 10 years. Little is known about its molecular mechanisms. In this study we overexpressed Nm23-H1 in H7721 cells and observed reduction of cell adhesion, migration and extension of actin stress fibers in cells stimulated by fibronectin (Fn).

**Methods:**

pcDNA3/Nm23-H1 was introduced into H7721 cells, and expression of Nm23-H1 was monitored by RT-PCR and western blot. Cell adhesion, actin extension and wound-induced migration assays were done on dishes coated with fibronectin. Phosphorylation of focal adhesion kinase (FAK) and total amount of integrin alpha5 and beta1 in Nm23-H1 transfected cells and control cells were measured by western blot. Flow cytometry was used to detect expression of surface alpha5 and beta1 integrin. N-glycosylation inhibitor tunicamycin was used to deglycosylate the integrin beta1 subunit.

**Results:**

Overexpression of *nm23-H1 *in H7721 cells reduced cell adhesion, migration and extension of actin stress fibers on dishes coated with Fn. Phosphorylation of FAK in Nm23-H1 transfected cells was also attenuated. Integrin alpha5 and beta1 gene messages were unaltered in *nm23-H1 *overexpressed cells as detected by RT-PCR. However, while cell surface integrin alpha5 was unchanged, surface expression of beta1 integrin was downregulated. Western blot also showed that the total amounts of integrin alpha5 and beta1 were unaltered, but the level of mature integrin beta1 isoform was decreased significantly. Furthermore, partially glycosylated precursor beta1 was increased, which indicated that the impaired glycosylation of integrin beta1 precursor might contribute to the loss of cell surface integrin beta1 in *nm23-H1 *overexpressed cells.

**Conclusion:**

These results suggest that by modulating glycosylation of integrin beta1, *nm23-H1 *down-regulates integrin beta1 subunit on cell surface and mediates intracellular signaling and subsequent suppression of the invasive process, including cell adhesion and migration.

## Introduction

Nonmetastatic protein 23 (Nm23) is a nucleoside diphosphate kinase that is conserved from bacteria to mammals [1]. Nm23 gene was isolated as a putative metastatic suppressor gene. Eight isotypes of the human NM23 gene (NM23-H1, NM23-H2, NM23-H3/DR-NM23, NM23-H4, NM23-H5, NM23-H6, NM23-H7, and NM23-H8) have been identified [2]. The *nm23-H1 *was firstly discovered in the members of this gene family [3], and demonstrated to have anti-metastatic properties in various models of human and animal cancer [4]. The gene is located on chromosome 17 q 21, which encodes an 18.5 kDa protein containing 166 amino acid residues with nucleoside diphosphate kinase, histidine kinase and serine autophosphorylation activities [5]. It is known that in many tumors high levels of *nm23-H1 *correlate with low degree of invasiveness. In addition, transfection of cancer cells with Nm23-H1 cDNA decreases their metastatic potential. However, the mechanism by which Nm23-H1 suppresses tumor metastasis is still poorly understood.

Tumor metastasis involves adhesive and migratory events in addition to proteolytic degradation of ECM [6], all of which require the continuous and coordinated formation and disassembly of adhesive structures. It involves stable attachment of a cell to the extracellular matrix at its leading edge which requires transmembrane receptors of the integrin family. Integrins are a super-family, and each of its members is a heterodimer composed of two noncovalently associated different subunits (α and β). At least 14 α and 8 β subunits have been discovered. The sizes of the α subunits are varied between 120~180 kDa, and those of β subunits are between 90~110 kDa. Most integrins are expressed on the surface of a wide variety of cells, and most cells express several integrins [7]. For example, α5 β1 integrin is a typical receptor of Fn [8] on HepG2 and Hep3B hepatocarcinoma cell lines [9]. ECM-integrin interaction generates intracellular signaling, which induces focal adhesion, actin cytoskeleton formation, cell migration, cell growth, and expression of various genes. To achieve correct cellular function through cell-matrix interaction, the ligation and clustering of integrins with their ligands need to be regulated in a number of ways. One way is to modulate the expression levels of integrins on cell surface. Another is to regulate the activity of integrins. It has been indicated that stimulation of β1 integrin by matrix protein initiates intracellular signaling pathways in many types of cells [10-12]. One of the initial events triggered by stimulation of β1 integrin is the association of its cytoplasmic domain with FAK, a cytosolic non-receptor tyrosine kinase, which leads to the tyrosine phosphorylation and activation of FAK [13,14]. Phosphorylated FAK is involved in the activation of many signal transduction molecules and affects several cellular biological behaviors [10,11,14].

In this report, we have studied cell adhesion, spreading and migration, as well as phosphorylation of FAK to fibronectin matrix in H7721 cell line transfected with Nm23-H1 cDNA. Furthermore, the expression of α5 and β1 integrin subunits in H7721 cells was examined, in an attempt to elucidate the molecular mechanism of suppressive effect of Nm23-H1 on cell invasion.

## Materials and methods

### Antibodies and Reagents

The human hepatocarcinoma H7721 cell line was obtained from the Institute of Cell Biology, Academic Sinica of China. RPMI 1640 and Geneticin (G418) were purchased from Invitrogen. Monoclonal antibody (mAb) of mouse anti-human Nm23-H1 was from Neomarkers Company. Monoclonal antibodies against human integrin α5 and β1, β-actin, rabbit polyclonal antibodies to human FAK, and Protein G PLUS agarose were from Santa Cruz Biotechnology Inc. Phosphotyrosine antibody (PT66), FITC-conjugated second antibodies, Fn and FITC-labeled phalloidin were purchased from Sigma. PVDF membrane was from Bio-Rad. TRIzol and AMV reverse transcriptase were from Promega. Other reagents, including Taq DNA polymerase, RNAase inhibitor, dNTP, oligo (dT)-18, ECL reagent were commercially available in China.

### Cell Culture, treatment and transfection

Cells were cultured at 37°C, 5% CO_2 _in RPMI-1640 medium containing 10% fetal calf serum. When Tunicamycin was used, its concentration was 2 μg/ml and the incubation time was 48 h.

The pcDNA3/Nm23-H1 plasmid was a kind gift of Prof Huili Chen in our department, constructed by Guo et al as described [15]. H7721 cells were transfected with pcDNA3/Nm23-H1 using lipofectamine. Stable transfectants, designated Nm23/H7721, were established by selection in 800 μg/ml G418 and were analyzed for Nm23-H1 expression by RT-PCR and western-blotting. One single clone which expressed the highest Nm23-H1 was chosen in this study. Empty vector control cells (Mock/H7721; pcDNA3 only) were generated by the same transfection and selection processes.

### Semiquantitative RT-PCR

Expression of *nm23-H1*, α5 and β1 mRNAs were determined by RT-PCR. The routine method of RT-PCR in our department was described previously [16]. Briefly, Total cell RNA was extracted with TRIzol and the complementary DNAs (cDNAs) were synthesized with oligo (dT)-18 primer and AMV reverse transcriptase from 3 μg total RNA. Then cDNA was amplified by Taq polymerase in 50 μl of reaction mixture containing 5 μl cDNA, 0.2 μM of the primer pair of *nm23-H1*, α5, β1 or β-actin (internal standard) according to the manual. From the 26th to 32nd cycle of PCR, 10 μl products were applied to agarose gel electrophoresis. The amplified DNA bands were scanned and the photographs were analyzed with NIH Image software. The semi-quantitative data were obtained by the intensity ratios of each PCR product to the β-actin. The primers of *nm23-H1*, *α5, β1 *and *β-actin *were synthesized by Sangon Company according to the reported sequences [17-19]

*nm23-H1 *F: 5'-ATGGCCAACTGTGAGCGTACC-3';

R: 5'-CATG TATTTCACCAGGCCGGC-3'. The product was 204 bp

*α5 *F: 5'-ACCAAGGCCCCAGCTCCATTAG -3';

R: 5'-GCCTCACACTGCAGGCTAAATG -3'. The product was 375 bp

*β1 *F: 5'-AACTTGATCCCTAAGTCAGCAGTAG-3';

R: 5'-ATCAGCAGTAATGCAAGGCC -3'. The product was 1200 bp

*β-actin *F: 5'-GATATCGCCGCGCTCGTCGTCGAC-3';

R: 5'-CAGGAAGGAAGGCTGGAAGAGTGC-3'. The product was 789 bp.

### Western blot analysis

Briefly, cells were homogenized in 0.1 M 2-(*N-*morpholino) ethanesulfonic acid (MES) buffer (pH 6.5)/150 mM NaCl/2% TritonX-100/25% glycerol/0.1 mg% leupeptin and pepstatin, and then centrifuged at 1000 μg at 4°C for 15 min. After determination of protein concentration, aliquots of 50 μg of protein samples were subjected to 10% SDS-PAGE and western blot according to the modified method of Knudsen et al. [20]. The membranes were blocked with 5% bovine serum albumin (BSA) in phosphate-buffered saline (PBS) overnight and treated with 1: 500 dilutions of different primary antibodies, followed by washing with 0.05% Tween-20/PBS for 3 times and incubation with 1: 500 dilution of HRP labeled secondary antibody for further 3 h. Then the membrane was washed again and stained with ECL reagent. β-actin was used as loading control and stained with 1: 800 dilution of primary antibody and 1: 500 dilution of HRP-labeled secondary antibody. Protein bands were quantified with densitometric analysis. Expression of each protein was calculated by the ratio of the intensity of this protein to that of β-actin.

### Assay of cell adhesion to Fn

Cell adhesion experiment was carried out according to the methods described by Busk et al [21]. In brief, the wells of culture plate were coated with 0.1 ml of different concentrations of Fn. In addition, 1 mg/ml poly-L-lysine and 1% BSA were coated for 2 wells each as maximal and minimal adhesion controls respectively. The plate was incubated at 37°C for 1 h, and blocked by 1% BSA at 37°C for 0.5 h after washing. Cells (1 × 10^5^) were added to each coated well and incubated for 2 h at 37°C, followed by staining with crystal violet after two washing, then the absorbance (Abs) at 595 nm was measured. Cell adhesion to the coated wells was calculated following a formula described in previous study [15]. The data were expressed as the mean of triplicate wells.

### Immunofluorescence Staining of Actin Filaments

Glass coverslips were coated with fibronectin as described above. Cells were plated onto the coverslips in 35-mm dishes and cultured for 24 h. Then they were fixed with 3.7% paraformaldehyde in PBS for 10 min and permeabilized with 0.5% Triton X-100 and 4% paraformaldehyde in PBS for 5 min. Actin filaments were stained with FITC-labeled phalloidin.

### Wound-induced Migration Assays

Wound-induced migration assay was performed as described elsewhere [22]. Cells (2 × 10^5 ^cells/well) were plated onto 12-well plastic plates coated with Fn (10 μg/ml) and cultured for 24 h. Then, subconfluent monolayers of the cells were scraped with a plastic pipette tip and washed with Hanks' solution twice, and the medium was replaced with serum-free RPMI-1640. The distance between migrating cell fronts was measured at 0 and 6 h after scraping.

### Detection of integrin subunits on cell surface by flow cytometry

Detection of cell surface integrin subunits was performed according to the method reported by Zhou et al [23]. Cells were dispersed in 2 mM EDTA in PBS and washed twice in PBS. Then 1 μ10^6 ^cells were incubated with monoclonal antibodies against α5 or β1 integrin subunits at a dilution of 1:100 in blocking buffer (1% BSA in PBS) for 45 min at 4°C. The cells were washed twice in blocking buffer, mixed with a 1:100 dilution of FITC-labeled goat anti-mouse IgG in blocking buffer, incubated for 30 min at 4°C, then the cells were washed again with PBS, suspended in 0.5 ml PBS and subjected to flow cytometry for fluorescence analysis. Integrin expression was determined to be the percentage of FITC-positive cells. The gate setting was determined by fluorescence intensity of the same cells stained with FITC-conjugated secondary antibody only.

### Determination of FAK autophosphorylation

Cells were plated onto culture dishes coated with 10 μg/ml fibronectin. Three hours after plating, the cells were washed twice with ice cold PBS, and the monolayer cells were lysed in 200 μl lysis buffer(50 mM pH7.4 HEPES/150 mM NaCl/100 mM NaF/1 mM MgCl2/1.5 mM EGTA/1% Nonidet P-40/10 μg/ml leupeptin and pepstatin, 1 mM PMSF). Cell lysate containing 500 μg protein (determined by Lowry's method) was incubated with 2 μg monoclonal antibody specific for FAK at 4°C for 1 h. Then 20 μl Protein G PLUS agarose suspension was added, and the sample was further incubated at 4°C for 3 h to immuno-precipitate FAK. Immuno-precipitated FAK was divided into two parts and subjected to 8% SDS-PAGE and western blot as described above. The membranes were probed with 1:1000 dilution of mouse monoclonal phosphotyrosine antibody (PT66) or 1: 500 dilution of FAK antibody, followed by incubation with 1: 500 dilution of HRP labeled second antibody. The color was developed with ECL reagent. The tyrosine phosphorylation (Tyr p) of FAK was calculated from the ratio of staining intensity of Tyr p to that of FAK.

### Statistical analysis

Values were expressed as mean ± SD. Statistical significance was determined with SPSS 10.0. Results were evaluated by Student's t tests. P < 0.05 and p < 0.01 were considered statistically significant and very significant respectively.

## Result

### Characterization of Nm23-H1 transfected cells

**Figure 1:**
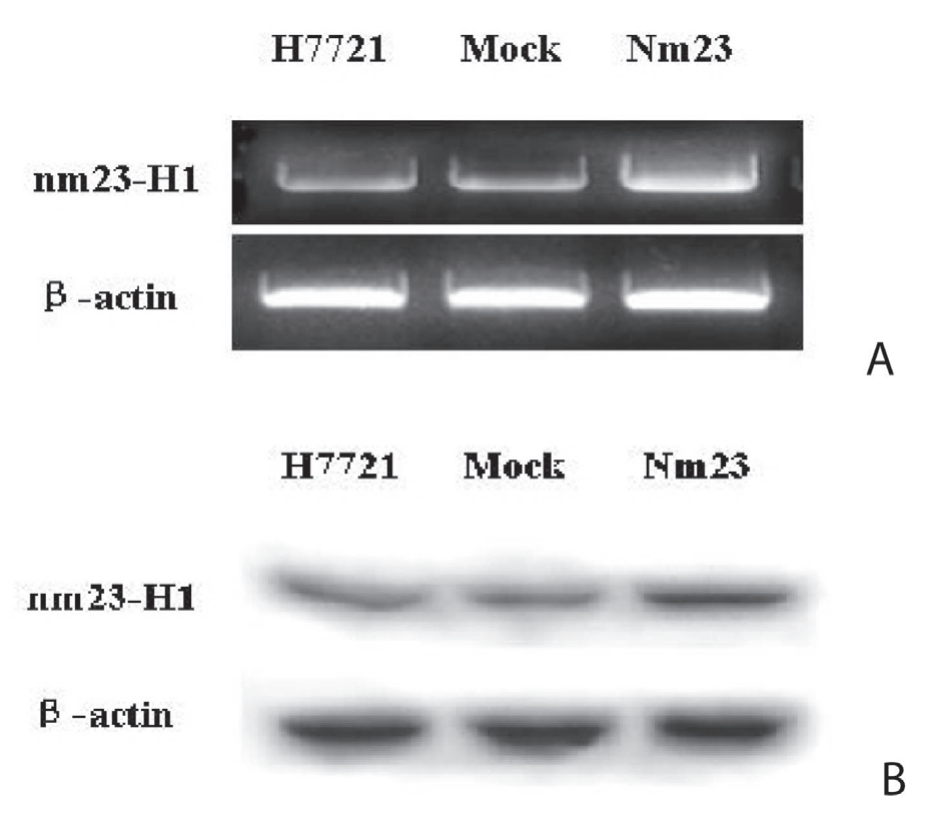
**Characterization of pcDNA3/Nm23-H1 transfected cells**. A. RT-PCR profiles of *nm23-H1 *mRNA in mock and pcDNA3/Nm23-H1 transfected cells. B. Western blot profiles of Nm23-H1 expression in mock and pcDNA3/Nm23-H1 transfected cells. Mock: H7721 cells transfected with pcDNA3 vector; Nm23: H7721 cells transfected with pcDNA3/Nm23-H1. The experimental procedures of RT-PCR and Western blot were described in the "Methods". Three independent experiments of A and B were performed and the results were reproducible.

Expression of Nm23-H1 was monitored by RT-PCR and western blot. In Nm23-H1 transfected cells, mRNA level of *nm23-H1 *was increased significantly when compared with that in mock-transfected cells. The ratio of *nm23-H1 *mRNA in Mock/H7721 to that in Nm23/H7721 was 1:2.94 ± 0.58 (p < 0.01). Meanwhile, the expression level of *nm23-H1 *between mock and wild H7721 cells showed no significant difference (Fig 1A). The western blot result was similar to that of RT-PCR with a ratio of Nm23/H7721 over Mock/H7721 Nm23-H1 level of 2.16 ± 0.37 (p < 0.01) (Fig 1B). These data indicates a successful transfection of H7721 cells with Nm23-H1.

### Alteration of cell adhesion, cytoskeleton formation and migration in cells transfected with *nm23-H1 *cDNA

**Figure 2:**
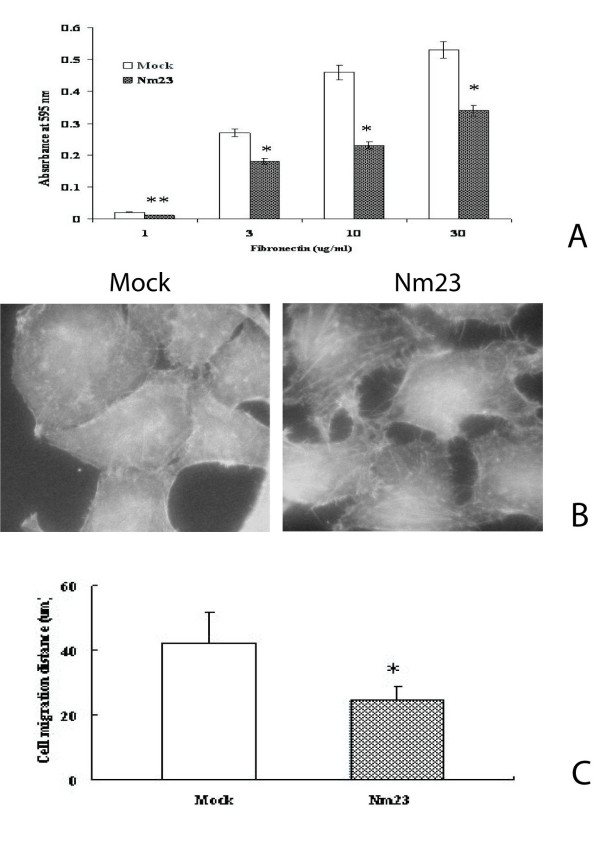
**Effect of Nm23-H1 overexpression on cell adhesion, cytoskeleton formation and migration to Fn**. A: Cell adhesion to fibronectin. *: p < 0.05 (n = 3). B: Cell cytoskeleton formation on fibronectin (× 100).C: Wound-induced migration assay. *: p < 0.01 (n = 20) Mock, Nm23: Same as Fig. 1. The experiment procedure was described in the "Methods".

Adhesion of mock and Nm23-H1 transfected cells to Fn was Fn concentration-dependent. However, the adhesion of the Nm23-H1 transfected cells to Fn was decreased in all concentrations tested as compared with the mocked cells tranfected with pcDNA3 vector (p < 0.05) (Fig. 2A).

Actin filaments were visualized with FITC-labeled phalloidin staining 24 hrs after cells being plated onto dishes coated with fibronectin. Fig. 2B showed mock-transfected cells formed well-developed actin stress fibers in ordered, compact and clear-cut structure with undisturbed edges. In contrast, Nm23-H1 transfected cells was disturbed and failed to form a complete cytoskeleton on fibronectin-coated dish.

As shown in Fig. 2C, cell migration was also decreased in Nm23-H1 transfected cells when compared with the mock-transfected cells (p < 0.01).

Taken together, these results are consistent with the conclusion that increased Nm23-H1 expression changed cell adhesion and migration to Fn.

### Effect of Nm23-H1 on expressions of integrin subunits on cell surface

**Figure 3:**
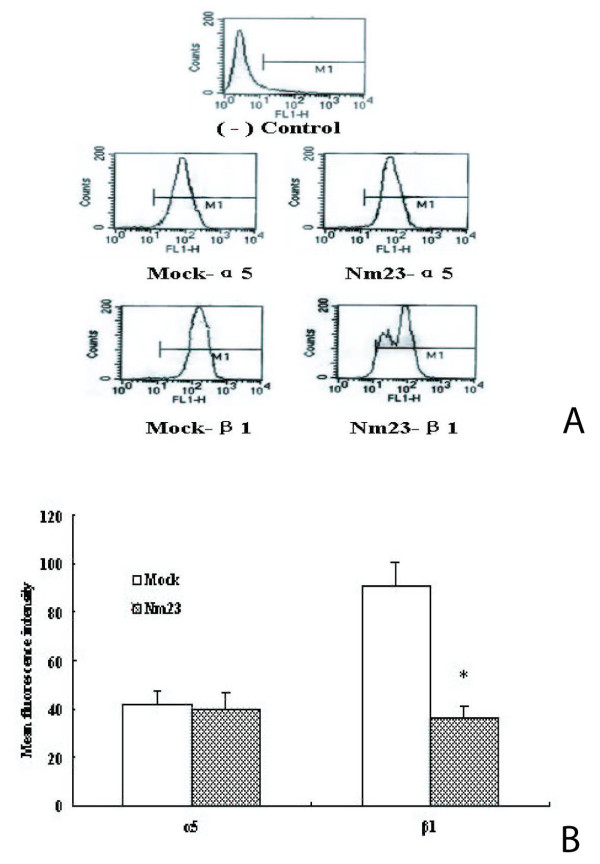
**Flow-cytometric analysis of α5 and β1 integrin subunits expression on cell surface after transfected with *nm23-H1 *cDNA**. A: Fluorescence activated cell spectra (FACS) of surface α5 and β1 integrin subunits. (-) Control: Sample without addition of primary antibody. B: Quantification of surface α5 and β1 integrin subunits, The data were expressed as the mean fluorescence Intensity (MFI) ± S.D. from 3 independent experiments. *: p < 0.01 compared to "Mock". Mock, Nm23: Same as Fig.1. The experiment procedure was described in the "Methods".

Given overexpression of Nm23-H1 impaired cell binding to Fn, it was important to determine if cell surface α5β1 integrin levels were altered. Fig 3A,B showed that the expression of β1 integrin subunit was down regulated to 39.6 ± 5.1% of the "Mock" level in Nm23/H7721 cells (p < 0.01). However, the expression of α5 subunit was unaltered on Nm23/H7721 cells compared with the Mock/H7721 cells.

### Expression of integrin subunit mRNAs in cells transfected with Nm23-H1

**Figure 4:**
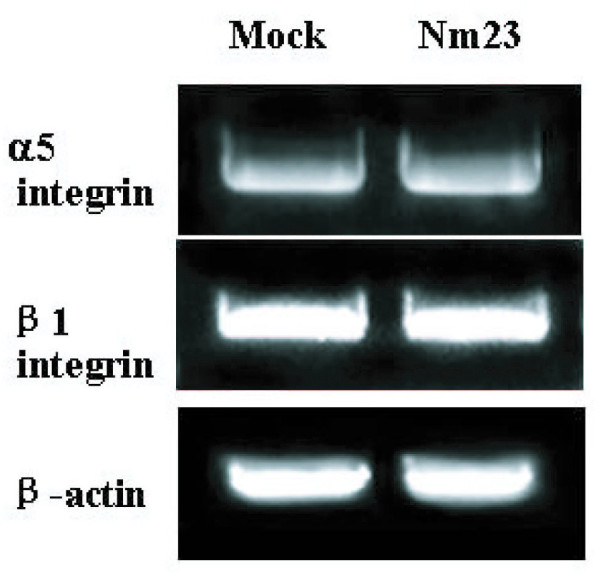
**RT-PCR analysis of α5 and β1 integrin subunits after transfected with *nm23-H1 *cDNA**. Mock, Nm23: Same as Fig.1. The experiment procedure was described in the "Methods".

Surface expression of integrin subunits was mainly regulated at transcriptional and post-transcriptional levels. In order to elucidate the mechanism of how Nm23-H1 regulates the expression of cell surface integrin subunits, we determined the mRNA levels of integrin subunits by RT-PCR. We found that mRNA levels of α5 and β1 subunit were not changed in Nm23/H7721 cells (Fig. 4). This data suggested that the decrease of cell surface integrin β1 subunit was not affected by transcriptional regulation.

### Altered glycosylation integrin subunit in cells transfected with Nm23-H1

**Figure 5:**
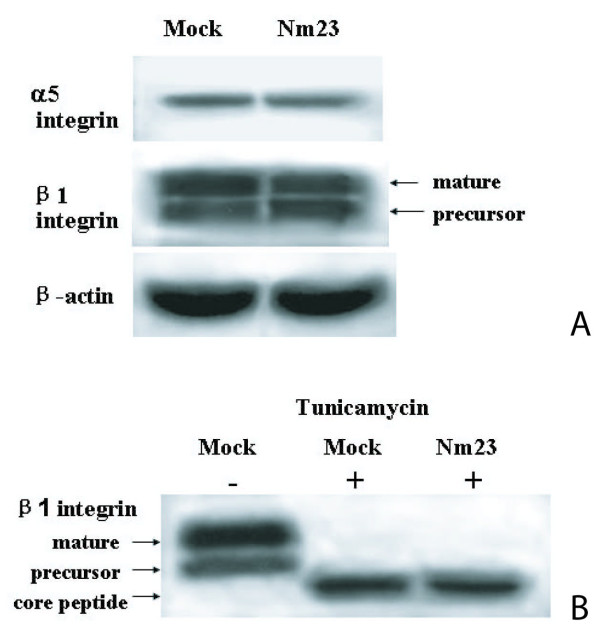
**Western blot analysis of α5 and β1 integrin subunits after transfected with *nm23-H1 *cDNA**. A: Western blot profiles of α5 and β1 integrin subunits expression in mock and pcDNA/Nm23-H1 transfected cells. B: Expression of β1 integrin subunits in cell treated with tunicamycin. Mock, Nm23: Same as Fig.1. The experiment procedure was described in the "Methods". Three independent experiments of A and B were performed and the results were reproducible.

To further study whether the decrease of integrin β1 subunits on cell surface was due to post-transcriptional regulation, we compared the total expression level of cellular β1 subunit by western blotting. As previously reported, two bands are typically observed in western blots of β1 integrin [24], namely a 115 kD partially glycosylated precursor and a 130 kD fully glycosylated mature form. It was very interesting to find that the total amount of β1 subunit was also unaltered in Nm23/H7721 cells, but the ratio of mature to precursor integrin isoforms was decreased significantly, being 1:1.21 ± 0.39 in Nm23/H7721 cells compared with 1:0.33 ± 0.12 in Mock cells (Fig 5A). This result suggested that overexpression of Nm23-H1 did not change total expression levels of β1 integrin. Instead, Nm23-H1 modulated the posttranslational processing of β1 integrin.

To further demonstrate that the alterated expression of mature β1 subunit was due to aberrant glycosylation, rather than other post-transcriptional regulation, we treated the cells with tunicamycin, an N-glycosylation inhibitor, and observed the deglycosylated form of β1 subunit. As shown in Fig. 5B, both Nm23/H7721 and Mock/H7721 cells only showed one band of about 90 kD crossed with intergrin β1 subunit antibody. Their size corresponded to the completely deglycosylated core peptide of the β1 subunit and their levels were almost equal. So these results indicated that the reduction of cell surface integrin β1 subunits in cells transfected with Nm23-H1 might be due to the changes of glycosylation.

### Effect of Nm23-H1 overexpression on the phosphorylation of FAK

**Figure 6:**
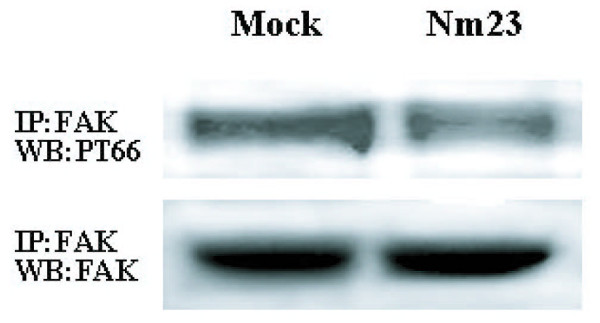
**Phophorylation of FAK in mock and pcDNA/Nm23-H1 transfected cells**. Mock, Nm23: Same as Fig.1. The experimental procedures of immuno-precipitation and Western blot were described in the "Methods". Three independent experiments were performed and the results were reproducible.

FAK is associated with the intracellular domain of integrin β subunit and involved in signaling transduction for cell adhesion and migration [25]. We tested whether Nm23-H1 overexpression affected phosphorylation of FAK on cells stimulated with fibronectin. As shown in Fig. 6, tyrosine autophosphorylation of FAK in Nm23-H1 transfected cells was decreased to 32.2 ± 6.4% (p < 0.01) compared with Mock cells.

## Discussion

Metastasis suppressor genes have contributed to our understanding of the metastasis process. They represent valuable therapeutic targets. Most evidences of metastasis suppressive activity were shown by transfection experiments using tumor cell line in which a low/mid-expressing metastasis suppressive gene is overexperessed. To date, seven metastasis suppressor genes have been confirmed--*nm23, Kiss 1, Kail, Brms1, E-cadherin, Maspin, and MKK4 *[26]. The antimetastatic effect of Nm23 has been an enigma for more than 10 years, but little is known about the molecular mechanisms underlying its role in cell physiology. A number of described data suggest that Nm23 directly and/or indirectly interferes with cell/extracellular matrix machinery [27].

Previous studies suggested that hepatocarcinoma-derived cells could be good models for the study of the molecular mechanisms involved in *nm23 *action [28]. To investigate the role of Nm23-H1 in tumor metastasis suppression and its possible mechanism, we established Nm23-H1 overexpressed hepatocarcinoma H7721 cell lines to determine their biological characteristics. In present study, we demonstrated that the overexpression of *nm23-H1 *in H7721 cells induced a marked decrease in cell's adhesive capacity, reorganization of actin stress fibers and motility on dishes coated with fibronectin. These findings were in agreement with the results that *nm23-H1 *had an inhibitory effect on cell migration.

As described before, α5β1 integrin is a typical receptor of Fn. Our data showed that expression of surface β1 integrin was downregulated in Nm23/H7721 cells, while the α5 integrin was unchanged. These results suggested that the ability of metastasis suppression by *nm23-H1 *might be partially due to the lower expression of β1 integrin. It was reported that the expression of β1 integrin was upregulated after transfection with a plasmid encoding DR-*nm23 *isoform in neuroblastoma cells, and this was correlated with an increase in cell adhesion on collagen type I [29]. By contrast, our results showed that the effect of *nm23-H1 *on the expression level of β1 integrin and the roles of β1 integrin has either a facilitatory or an inhibitory effect on cell migration. This discrepancy may be due to the different cell lines and ECM components used in these studies.

Furthermore, we have investigated the potential mechanism of reduced surface expression of integrin β1 subunit in Nm23-H1 overexpressing cells. Initially we speculated the changes of integrin β1 expression on cell surface were due to the regulation of gene transcriptional level by Nm23-H1. Nm23-H1 is a versatile kinase that can phosphorylate nucleoside diphosphate molecules and histidine residues on target proteins as well as autophosphorylate itself on at least two specific serine residues [30]. Given their characteristically broad substrate specificities, they can alter expression of many downstream genes [31,32]. It was surprising to find that expression of β1 integrin mRNA level was unaltered in Nm23/H7721 cells. This data suggested that the reduction of integrin β1 expression on cell surface was probably due to post-transcriptional mechanism.

Protein glycosylation is an important event for post-transcriptional regulation that contributes to protein maturity. Integrin β1 subunit is a transmembrane glycoprotein. Intriguingly, the β1 integrin may be well positioned for regulation by glycosylation. Unlike other integrin subunits, partially glycosylated β1 integrin precursors also form a stable pool within the endoplasmic reticulum [33-36]. The cell, therefore, may be able to direct the expression of a variant glycosylated species by recruiting precursors from the ER.

How the β1 integrin traffics from ER to Golgi is still unclear. However, this transition indicates a potential target for regulation of β1 integrin expression on cell surface. Our findings in Fig 5A showed that total amount of β1 subunit in Nm23/H7721 cells did not change, which was consistent with the results obtained by RT-PCR. But, the level of mature integrin isoform was decreased significantly, while the level of partially glycosylated precursor was increased. It suggests that the expression of Nm23-H1 affects the glycosylation of integrin β1 precursor and the altered glycosylation of integrin β1 may contribute to the loss of cell surface integrin β1 in Nm23/H7721 cells. In previous studies by others, it was demonstrated that Nm23-H1 could down regulate the transcription of many glycosyltransferase genes, including *GnT-V*, α1,3*FucTs *and *ST3Gals *and that they were correlated with anti-metastasis effect in tumor cells [15,37]. Accumulating evidence indicates that β1 integrin is an important target for GnT-V and ST6Gal. Therefore, it may be concluded that transfection of Nm23-H1 cDNA down regulates some key glycosyltransferase genes and then interferes the protein post-translational modification. In consequence, the glycosylation of β1 integrin precursor is impaired, leading to the loss of cell surface β1 integrin. However, the detailed mechanisms need to be further investigated. The mechanisms of regulating integrin-stimulated cell migration are very complex and the activation of tyrosine kinases plays an important role in these events [4]. Emerging evidence supports the important role of FAK PTK in these processes. FAK activation has been linked to integrin clustering and is considered as a critical step in the initiation of cell migration. In cultured cells, overexpression of FAK can increase Fn-stimulated cell motility and this activity depends upon the integrity of the FAK Tyr-397 autophosphorylation site [38,39]. Our result showed that Nm23-H1 seemed to have no effect on the expression of FAK in H7721 cells, while it decreased the tyrosine phosphorylation of FAK, an important event in integrin-mediated signaling.

Together, our results suggested that overexpression of Nm23-H1 in H7721 cells interfered the expression of some glycosyltransferases, impaired glycosylation of β1 integrin precursor, and in turn down-regulated integrin β1 expression on cell surface, resulting in the reduction of cells interacted with fibronectin, which abrogated intracellular signals that mediate focal adhesion, actin cytoskeleton formation and cell migration. It is the first evidence that links *nm23-H1 *to the glycosylation of integrin β1, which is interesting for further study.

## Competing interests

The authors declare that they have no competing interests.

## Authors' contributions

SS and XB formulated the research protocol and carried out the follow up of participants. HM and LX participated in the design of the study and performed the statistical analysis. WQ participated in the design of the study, and the execution of the study protocol. All authors read and approved the final manuscript.

## References

[B1] Tee YT Chen GD Lin LY Ko JL Wang PH Nm23-H1: a metastasis-associated gene Taiwan J Obstet Gynecol 2006 45 107 113 10.1016/S1028-4559(09)60206-0 17197349

[B2] Lacombe ML Milon L Munier A Mehus JG Lambeth DO The human Nm23/nucleoside diphosphate kinases J Bioenerg Biomembr 2000 32 247 58 10.1023/A:1005584929050 11768308

[B3] Gilles AM Presecan E Vonica A Lascu I Nucleoside diphosphate kinase from human erythrocytes J Biol Chem 1991 266 8784 8789 1851158

[B4] Steeg PS Ouatas T Halverson D Palmieri D Salermo M Metastasis suppressor genes: basic biology and potential clinical use Clin Breast Cancer 2003 4 51 62 10.3816/CBC.2003.n.012 12744759

[B5] De La Rosa A Williams RL Steeg PS Nm23/nucleoside diphosphate kinase: toward a structural and biochemical understanding of its biological functions BioEssays 1995 17 53 62 10.1002/bies.950170111 7702594

[B6] Liotta LA Cancer cell invasion and metastasis Sci Am 1992 266 54 59 62-63 10.1038/scientificamerican0292-54 1373003

[B7] Hynes RO Integrin: Versarility, modulation, and signaling in cell adhesion Cell 1992 69 11 25 10.1016/0092-8674(92)90115-S 1555235

[B8] Zheng MZ Fang H Hakomori S Functional role of N-glycosylation in α5 β1 integrin receptor J Biol Chem 1994 269 12325 12331 7512965

[B9] Nejjari M Hafdi Z Dumortier J Bringuier AF Feldmann G Scoazec JY alpha 6 beta 1 integrin expression in hepatocarcinoma cells: Regulation and role in cell adhesion and migration Int J Cancer 1999 83 518 525 10.1002/(SICI)1097-0215(19991112)83:4<518::AID-IJC14>3.0.CO;2-Q 10508489

[B10] Clark EA Hynes RO Keystone symposium on signal transduction by cell adhesion receptors Biochim Biophys Acta 1997 1333 R9 16 9426206 10.1016/s0304-419x(97)00028-0

[B11] Giancotti FG Ruoslahti E Integrin signaling Science 1999 285 1028 32 10.1126/science.285.5430.1028 10446041

[B12] Basson MD An intracellular signal pathway that regulates cancer cell adhesion in response to extracellular forces Cancer Res 2008 68 2 4 10.1158/0008-5472.CAN-07-2992 18172287

[B13] Kornberg LJ Earp HS Turner CE Prockop C Juliano RL Signal transduction by integrins: increased protein tyrosine phosphorylation caused by clustering of beta 1 integrins Proc Natl Acad Sci USA 1991 88 8392 8396 10.1073/pnas.88.19.8392 1717976 PMC52514

[B14] Schlaepfer DD Hauck CR Sieg DJ Signaling through focal adhesion kinase Prog Biophys Mol Biol 1999 71 435 78 10.1016/S0079-6107(98)00052-2 10354709

[B15] Guo HB Liu F Zhao JH Chen HL Down-regulation of N- acetylglucosaminyl- transferase V by tumorigenesis- or metastasis-suppressor gene and its relation to metastatic potential of human hepatocarcinoma cells J Cell Biochem 2000 79 370 385 10.1002/1097-4644(20001201)79:3<370::AID-JCB30>3.0.CO;2-Z 10972975

[B16] Guo P Zhang Y Shen Z Zhang X Chen H Effect of *N*-acetylglucosaminyltransferase V on the expressions of other glycosyl- transferases FEBS lett 2004 562 93 96 10.1016/S0014-5793(04)00188-7 15044007

[B17] Yokoyama A Okabe-Kado J Wakimoto N Kobayashi H Sakashita A Maseki N Nakamaki T Hino K Tomoyasu S Tsuruoka N Motoyoshi K Nagata N Honma Y Evaluation by Multivariate Analysis of the Differentiation Inhibitory Factor nm23 as a Prognostic Factor in Acute Myelogenous Leukemia and Application to Other Hematologic Malignancies Blood 1998 91 1845 1851 9490665

[B18] Cai T Lei QY Wang LY Zha XL TGF-β1 modulated the expression of α5β1 integrin and integrin-mediated signaling in human hepatocarcinoma cells Biochem Biophys Res Commun 2000 274 519 525 10.1006/bbrc.2000.3177 10913370

[B19] Kudo T Ikehara Y Togayachi A Morozumi K Watanabe M Nakamura M Nishihara S Narimatsu H Up-regulation of a set of glycosyltransferase genes in human colorectal cancer Lab Invest 78 797 811 9690558

[B20] Knudsen KE Arden KC Cavenee WK Multiple G1 regulatory element control the androgen-dependent proliferation of prostate carcinoma cells J Biol Chem 1998 273 20213 20222 10.1074/jbc.273.32.20213 9685369

[B21] Busk M Pylela R Sheppard D Characterization of integrin alpha V beta 6 as a fibronectin-binding protein J Biol Chem 1992 267 5790 5796 1532572

[B22] Goodman SL Vollmers HP Birchmeier W Control of cell locomotion: perturbation with an antibody directed against specific glycoproteins Cell 1985 41 1029 1038 10.1016/S0092-8674(85)80083-0 2408757

[B23] Zhou GF Feng Y Cao LH Zha XL Over-expression of integrin alpha5beta1 in human hepatocarcinoma cell line suppresses cell proliferation in vitro and tumorigenicity in nude mice Mol Cell Biochem 2000 207 49 55 10.1023/A:1007034012642 10888226

[B24] Argraves WS Suzuki S Arai H Thompson K Pierschbacher MD Ruoslahti E Amino acid sequence of the human fibronectin receptor J Cell Biol 1987 105 1183 90 10.1083/jcb.105.3.1183 2958481 PMC2114793

[B25] Clark EA Brugge JS Integrins and signal transduction pathways: the road taken Science 1995 268 233 239 10.1126/science.7716514 7716514

[B26] Yoshida BA Sokoloff MM Welch DR Rinker-Schaeffer CW Metastasis-suppressor genes: a review and perspective on an emerging field J Natl Cancer Inst 2000 92 1717 30 10.1093/jnci/92.21.1717 11058615

[B27] Fournier HN Albiges-Rizo C Block MR New insights into Nm23 control of cell adhesion and migration J Bioenerg Biomembr 2003 35 81 87 10.1023/A:1023450008347 12848345

[B28] Boissan M Lacombe ML Nm23/NDP kinases in hepatocellular carcinoma J Bioenerg Biomembr 2006 38 169 75 10.1007/s10863-006-9031-4 16944304

[B29] Amendola R Martinez R Negroni A Venturelli D Tanno B Calabretta B Raschella G DR-nm23 gene expression in neuroblastoma cells: relationship to integrin expression, adhesion characteristics, and differentiation J Natl Cancer Inst 1997 89 1300 1310 10.1093/jnci/89.17.1300 9293921

[B30] Steeg PS Wagner PD Nm23 and tumor metastasis: biochemical and translational advances Adv Oncol 1997 13 3 9

[B31] Steeg PS Horak CE Miller KD Nm23/NDP kinases in hepatocellular carcinoma Clin Cancer Res 2008 14 5006 12 10.1158/1078-0432.CCR-08-0238 18698018 PMC2730725

[B32] Postel EH Berberich SJ Rooney JW Kaetzel DM Human NM23/nucleoside diphosphate kinase regulates gene expression through DNA binding to nuclease-hypersensitive transcriptional elements J Bioenerg Biomembr 2000 32 277 284 10.1023/A:1005541114029 11768311

[B33] Heino J Ignotz RA Hemler ME Crouse C Massague J Regulation of cell adhesion receptors by transforming growth factor-beta. Concomitant regulation of integrins that share a common beta 1 subunit J Biol Chem 1989 264 380 388 2491849

[B34] Lenter M Vestweber D The integrin chains beta 1 and alpha 6 associate with the chaperone calnexin prior to integrin assembly J Biol Chem 1994 269 12263 12268 8163531

[B35] Akiyama SK Yamada KM Biosynthesis and acquisition of biological activity of the fibronectin receptor J Biol Chem 1987 262 17536 17542 2961737

[B36] Jaspers M de Strooper B Spaepen M van Leuven F David G van den Berghe H Cassiman JJ Post-translational modification of the beta-subunit of the human fibronectin receptor FEBS Lett 1988 231 402 406 10.1016/0014-5793(88)80859-7 2966078

[B37] Duan LL Guo P Zhang Y Chen HL Regulation of metastasis-suppressive gene Nm23-H1 on glycosyl-transferases involved in the synthesis of sialyl Lewis antigens J Cell Biochem 2005 94 1248 1257 10.1002/jcb.20346 15696547

[B38] Gates RE King LE Jr Hanks SK Nanney LB Potential role for focal adhesion kinase in migrating and proliferating keratinocytes near epidermal wounds and in culture Cell Growth Differ 1994 5 891 899 7986754

[B39] Cary LA Chang JF Guan JL Stimulation of cell migration by overexpression of focal adhesion kinase and its association with Src and Fyn J Cell Sci 1996 109 1787 94 8832401 10.1242/jcs.109.7.1787

